# Stereotaxic Adeno-associated Virus Injection and Cannula Implantation in the Dorsal Raphe Nucleus of Mice

**DOI:** 10.21769/BioProtoc.2549

**Published:** 2017-09-20

**Authors:** Patrícia A. Correia, Sara Matias, Zachary F. Mainen

**Affiliations:** 1 Champalimaud Research, Champalimaud Centre for the Unknown, Lisbon, Portugal

**Keywords:** Adeno-associated virus, Optogenetic probes, Serotonin, Dorsal raphe nucleus, Stereotaxic surgery, Optical cannula

## Abstract

Optogenetic methods are now widespread in neuroscience research. Here we present a detailed surgical procedure to inject adeno-associated viruses and implant optic fiber cannulas in the dorsal raphe nucleus (DRN) of living mice. Combined with transgenic mouse lines, this protocol allows specific targeting of serotonin-producing neurons in the brain. It includes fixing a mouse in a stereotaxic frame, performing a craniotomy, virus injection and fiber implantation. Animals can be later used in behavioral experiments, combined with optogenetic manipulations (Dugué *et al*., 2014; Correia *et al*., 2017) or monitoring of neuronal activity (Matias *et al*., 2017).

The described procedure is a fundamental step in both optogenetic and fiber photometry experiments of deep brain areas. It is optimized for serotonin neurons in the DRN, but it can be applied to any other cell type and brain region. When using transgenic mouse lines that express functionally relevant levels of optogenetic tools or reporter lines, the virus injection step can be skipped and the protocol is reduced to the cannula implantation procedure.

## Background

With the advent of optogenetic methods, the use of optical fibers and genetically encoded probes to manipulate or monitor brain activity has rapidly expanded. Optogenetic tools are particularly useful to study neuromodulatory systems, as they are usually characterized by clusters of neurons located in deep brain regions, with long and wide projections to a multitude of brain areas. Virus injections and fiber cannula implantations have been previously described for diverse areas in the brain (*e.g*., ventral tegmental area [Tsai *et al*., 2009], locus coeruleus [Carter *et al*., 2010]).

Targeting the dorsal raphe nucleus (DRN, the main source of serotonin projections to the forebrain) can be complex, given its deep anatomical location below the aqueduct and the superior sagittal sinus. Using standard surgical procedures might cause extensive bleeding and low success rate, resulting in small sample sizes (Ranade and Mainen 2009; Cohen *et al*., 2015). Optogenetic studies targeting the DRN have been previously described (Dugué *et al*., 2014; Liu *et al*., 2014; Qi *et al*., 2014; McDevitt *et al*., 2014; Ogawa *et al*., 2014; Pollak Dorocic *et al*., 2014; Weissbourd *et al*., 2014; Miyazaki *et al*., 2014; Fonseca *et al*., 2015; Cohen *et al*., 2015; Li *et al*., 2016; Correia *et al*., 2017; Matias *et al*., 2017), but a detailed and efficient surgery protocol is lacking. Here we present a protocol to target viral transduction to serotonin-producing neurons in the DRN and perform optical fiber implantation with an angled approach, to avoid the superior sagittal sinus. When performing exclusively viral transduction, it is not essential to use an angled approach (Qi *et al*., 2014; McDevitt *et al*., 2014; Ogawa *et al*., 2014; Pollak Dorocic *et al*., 2014; Weissbourd *et al*., 2014). The protocol described here can be used for diverse optogenetic procedures, such as photostimulation, photoinhibition, or fiber photometry.

## Materials and Reagents

1. Surgical drape
2. Quartz pipettes (Quartz with filament OD 1.0 mm, ID 0.5 mm, 7.5 cm length) (Sutter Instrument, catalog number: QF100-50-7.5)
3. Petri dish
4. Parafilm (BRAND, catalog number: 701605)
5. Absorbable sponges (Spongostan Dental, Ferrosan Medical Devices) (Ethicon, catalog number: MS0005)
6. Cotton swabs (Henry Schein, catalog number: 100-6015)
7. Bone scraper (Fine Science Tools, catalog number: 10075-16)
8. Surgical blade (Swann Morton, catalog number: 0301)
9. Needles (30 G) (BD, catalog number: 304000)
10. Cleaning wipes (Kimwipes, Kimtech) (KCWW, Kimberly-Clark, catalog number: 34120)
11. Stitching kit and sutures (Vicryl) (Ethicon, catalog number: MPV494H)
12. SERT-Cre C57BL/6 mice (*Slc6a4^tm1(cre)Xz^*, THE JACKSON LABORATORY, catalog number: 014554) and WT C57BL/6 mice (littermates control)
13. Virus AAV2.9.EF1a.DIO.hChR2(H134R)-eYFP.WPRE.hGH (10^13^ GC/ml) for photostimulation or AAV2/1-Syn-Dio-GCaMP6s (10^13^ GC/ml) for fiber photometry (University of Pennsylvania Vector Core)
14. Sterile saline 0.9% NaCl (B. Braun Melsungen)
15. Isoflurane (4% induction and 0.5-1% for maintenance, Vetflurane, Virbac)
16. Analgesic (*e.g*., Dolorex, Butorphanol, http://www.dolorex.info/dolorex/dolorex.asp, 10 mg/ml, injectable solution, Intervet, Schering-Plough Animal Health)
17. Betadine 10% (Lainco S.A.)
18. Lidocaine 2% (Braun 20 mg/ml) (B. Braun Melsungen, catalog number: RVG 56836)
19. Eye ointment (*e.g.,* Vidisic, Carbomer 980, https://www.hpra.ie/img/uploaded/swedocuments/2122630.PA0555_006_001.6a418525-a1b3-46ca-af2c-402404b85680.000001Product%20leaflet%20approved%201.140331.pdf, Bausch & Lomb)
20. Distilled water
21. Gentamicin 0.3% (Sigma-Aldrich, catalog number: 48760)
22. Dental acrylic (Pi-Ku-Plast HP 36, Bredent, catalog numbers: 54000213 and 54000215)
23. Veterinary wound powder (Battle, catalog number: 2281)
24. Super Bond C&B (Sun Medical, catalog number: P021E/0A)

## Equipment

1. Anesthesia system for isoflurane (Matrx by Midmark, model: VIP 3000^®^)
2. Heating pad
3. Stereotaxic frame (KOPF INSTRUMENTS, model: Model 902)
4. Mouse adaptor for gas anesthesia (KOPF INSTRUMENTS, model: Model 923-B)
5. Electric clipper for cutting mouse hair (WAHL Clipper, model: 5540)
6. Pipette puller (Sutter Instruments, model: P-2000)
7. Scissors (Fine Science Tools, catalog number: 14088-10)
8. Fine tip forceps (Fine Science Tools, catalog number: 11242-40)
9. Suture scissors (Fine Science Tools, catalog number: 12001-13)
10. Scalpel handle (Fine Science Tools, catalog number: 10003-12)
11. Colibri retractor (Fine Science Tools, catalog number: 17000-02)
12. Sterile glass beaker
13. Dental drill (Midwest Tradition PB Handpieace Non-Fiber Optic, DENTSPLY International, catalog number: 790044)
14. Drill bits (CARBIDE BUR FG 1/4) (Henry Schein, catalog number: 101-7864)
15. Suction tool to aspirate viral solution into the pipette (Sigma-Aldrich, catalog number: A5177-5EA)
16. Picospritzer (Parker Hannafin, model: Picospritzer III)
17. Optical fiber (200 μm core diameter, 0.48 NA, 4-5 mm) housed inside a connectorized implant (M3, Doric lenses)
18. Zygomatic ear cups, serrated (KOPF INSTRUMENTS, model: Model 921)
19. Microscope (Leica Microsystems, model: Leica MZ6)

## Procedure

The surgery setup consists of a stereotaxic frame connected to a gas anesthesia system, situated on top of a surgery table. A heating pad covered by a surgical drape is placed on the stereotaxic frame, below the mouse mask (where the animal will be placed). The picospritzer for the virus injection is located on a shelf, close to the surgery table. The microscope is attached to the wall, allowing movements in different angles. The aseptic surgical field is the disinfected skin and exposed surgical wound. All materials necessary for surgery (including the dental drill) are within reach around the stereotaxic frame.

**Figure 1:**
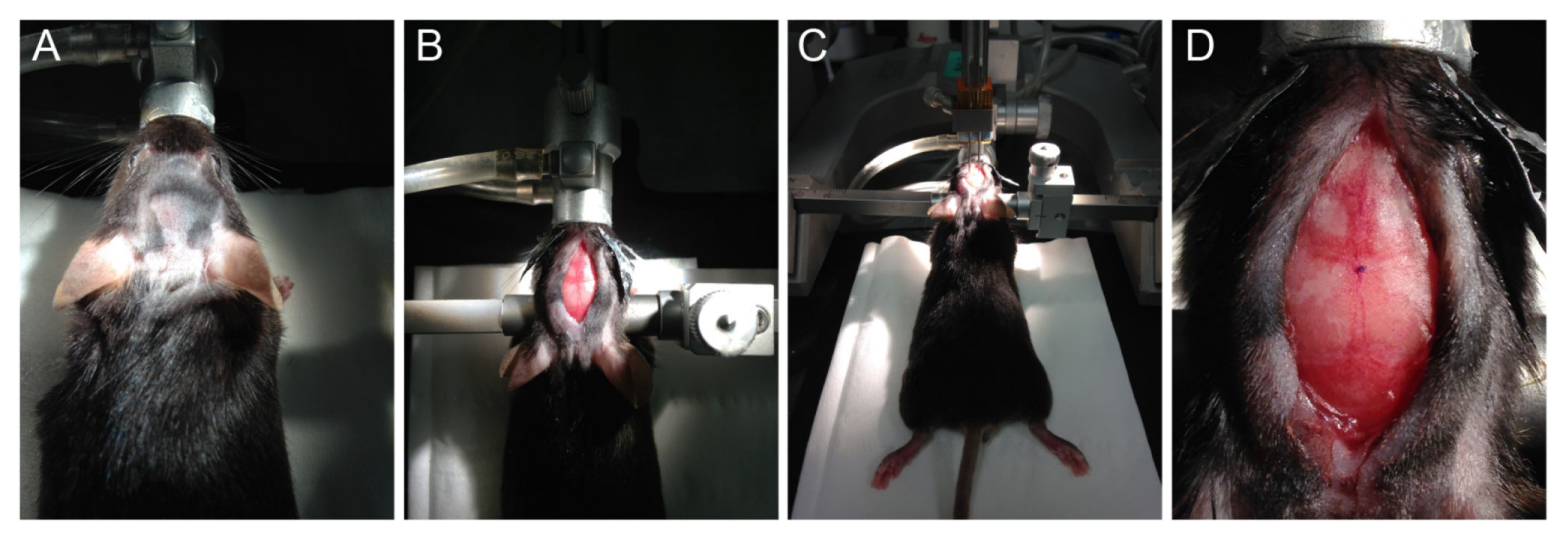
Mouse preparation for craniotomy. A. Shaved area of the mouse head; B. Mouse placed in the stereotaxic frame with zygomatic ear cups, depicting head incision with cleaned skull. C. Alignment of the skull, using two needles mounted on the stereotaxic holder. D. Super bond layer applied to the skull, exposing bregma mark.

**Figure 2:**
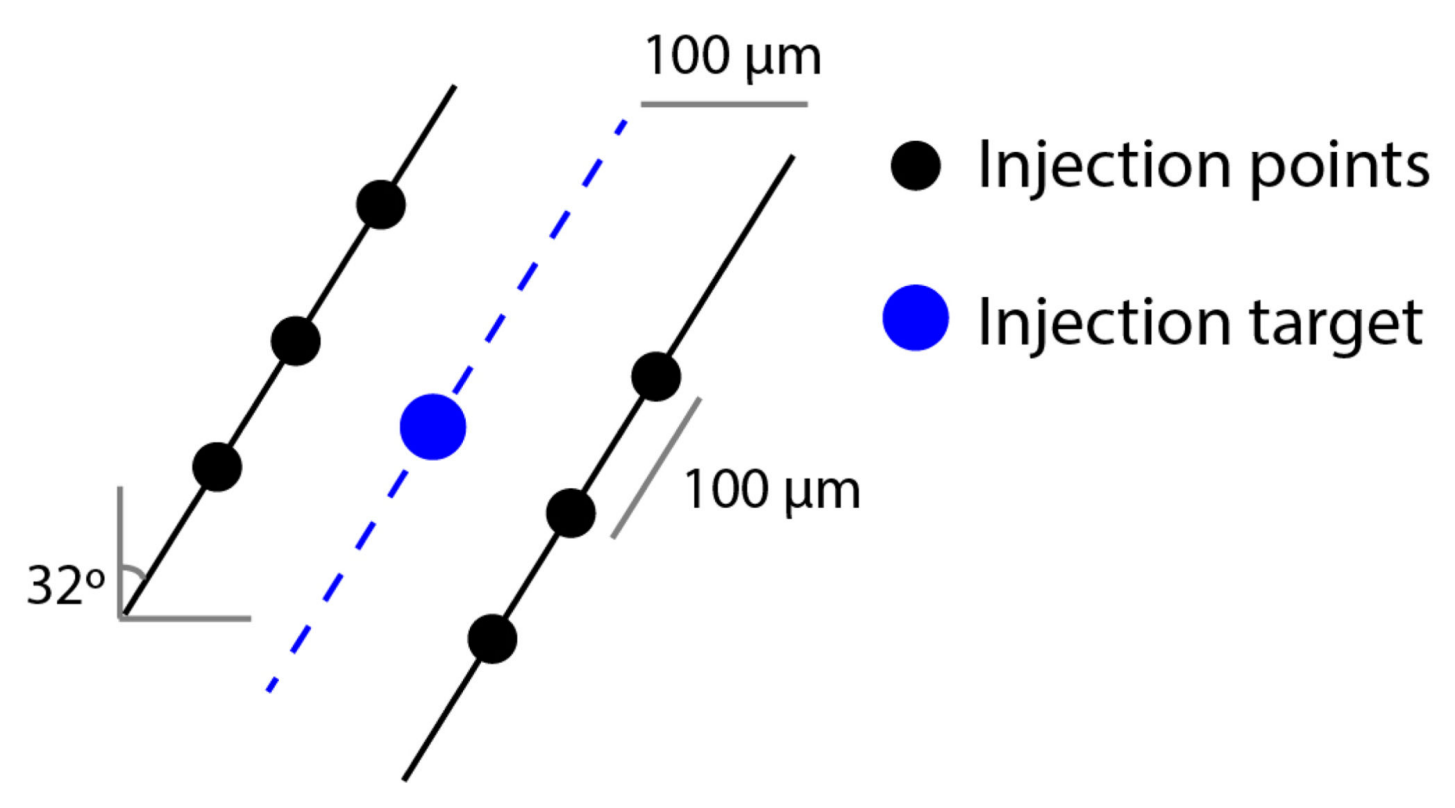
Virus injection in the dorsal raphe nucleus. Injection is performed with an angled approach (32°), in six different points (black circles) around the target (blue circle). We observed that the viral spread within the DRN is low (when compared with other brain areas), thus we use six points of injection to guarantee a wider spread of the viral particles within the DRN.

**Figure 3:**
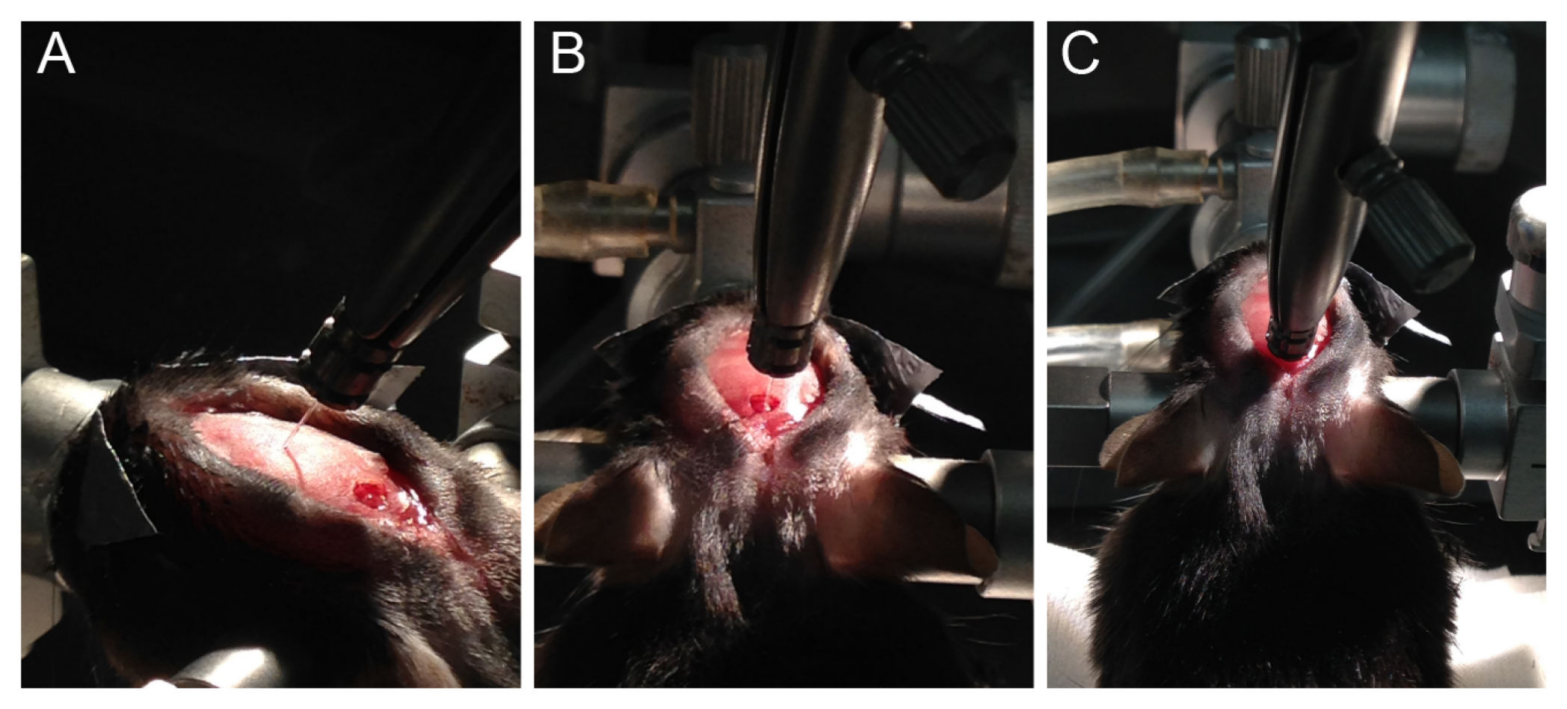
Optical fiber implantation in the DRN. A. Fiber mounted in stereotaxic holder and positioned in bregma; B. Fiber positioned on the surface of the brain, in the DRN; C. Fiber implanted in the dorsal-ventral position of the DRN.

- Preparation for surgery
  1. Get a glass pipette using the pipette puller and mark it in three locations (upper and lower limit of 1 μl total volume–5.9 mm in these pipettes–plus one mark half way).
  2. Set the right arm of the stereotaxic frame at 32° (the injection is performed with an angled approach from the back to avoid breaking the superior sagittal sinus).
  3. Check isoflurane level in the anesthesia system and fill it if needed.
  4. Place the Super Bond dispensing dish at 4 °C.
  5. Fill up one pipette with virus using the suction tool and store it in the fridge (place the pipette inside a Petri dish and cover with Parafilm).
  6. Prepare a 25 ml glass beaker with 10 ml saline and add small pieces of absorbable sponges.
  7. Turn on the heating pad (37 °C).
- Mouse preparation
  1. Weight the mouse (SERT-Cre or WT).
  2. Anesthetize mouse in the isoflurane induction chamber (4%, 1 L/min).
  3. Place animal in the anesthesia mask.
  4. Give analgesic (*e.g*., Dolorex 10 mg/ml, dilute 1:20 in saline and use 0.1 ml per 25 g of animal) subcutaneously, after anesthesia induction.
  5. Shave the head, from the eyes to behind the ears (Figure 1A).
  6. Place the mouse on the heating pad, fix it in the stereotaxic frame and adjust isoflurane to 0.5-1% while monitoring the mouse’s breathing rate.
  7. Anesthesia is confirmed by absence of a response to a toe pinch (monitor toe pinch response every 20 min during surgery).
  8. Use cotton swabs to clean the head with betadine, distilled water, and then betadine.
  9. Inject 0.1 ml Lidocaine under the surface of the scalp to provide local analgesia.
  10. Protect eyes from light: put eye ointment (*e.g*., Vidisic, 2 mg/ml) and cover them.
- Incision and craniotomy
  1. Make incision from anterior to posterior (between the eyes to back of the skull) (Figure 1B).
  2. Swab incision with a cotton swab dipped in saline.
  3. Scrape away tissues on top of skull with a scraper.
  4. Clean skull with a cotton swab dipped in distilled water.
  *Note: Steps C3-C4 are crucial for implantation. It is optional to use a Colibri retractor to expose the surgical field*.
  - If using zygomatic ear cups, align the skull (make it flat) in the anterior-posterior and medial-lateral axis using two needles mounted on a stereotaxic holder in the left arm (Figure 1C). In the case of ear bars, just focus on the anterior-posterior (bregma-lambda) alignment.
  - Estimate and mark bregma location.
  - Make sure skull is clean (without blood, fur or tissue) and dry (use a dry cotton swab to absorb any distilled water or blood).
  - Cut thin marks into bone with a scalpel (improves adhesion of super bond to the skull) and dry well the skull using a compressed air duster.
  - Get Super Bond container from fridge and prepare the mixture, following instructions (see Recipes).
  - Apply a thin layer of Super Bond on the skull, using the brush. It is crucial to leave bregma mark exposed (Figure 1D). Do not apply Super Bond on the skin.
  *Note: It is important to be fast performing steps C9-C10. If Super Bond is applied to the skin, clean it immediately, using forceps to gently remove it*.
  - Using one needle mounted on a stereotaxic holder in the right arm (32° angled), mark bregma and calculate target coordinates (DRN is -4.7 AP, -2.9 DV from bregma), using the following equation.
    $$\begin{array}{l} xB-x\;\text{bregma}\;\text{reading}\\ zB-z\;\text{bregma}\;\text{reading}\\ x_{t}-AP\;\text{position}\;\text{of}\;\text{the}\;\text{target}\;(e.g.-4.7\;mm)\\ z_{t}-DV\;\text{position}\;\text{of}\;\text{the}\;\text{target}\;(e.g.-2.9\;mm)\\ x_{f}-\text{final}\;AP\;\text{position}\;\text{of}\;\text{the}\;\text{target}\;(\text{considering}\;\text{angled}\;\text{approach})\\ z_{f}-\text{final}\;DV\;\text{position}\;\text{of}\;\text{the}\;\text{target}\;(\text{considering}\;\text{angled}\;\text{approach})\\ \alpha -\text{angle}\;(32{}^{\circ})\\ x_{f}=x_{B}+x_{t}+z_{t}\times \text{tan}\;\alpha \\ z_{f}=z_{B}+\frac{z_{t}}{\text{cos}\;\alpha } \end{array}$$
    *Note: This equation is used to calculate the corrected target coordinates after having a defined angle for the implantation. When no angle is used in the stereotaxic arm, it is enough to sum the coordinates of the target to the coordinates read in the stereotaxic frame while touching bregma. However, when using an angle, the target coordinates need to be corrected to account for such angle, using simple trigonometry*.
  - Mark target position.
  - Perform craniotomy around the target mark (drill through Super Bond) and remove the dura using a 30 G needle. If any bleeding occurs, use a cotton swab to clean the blood and use a piece of absorbable sponge to stop the bleeding.
  - Cover craniotomy with a wet sponge in saline.
- Virus injection
  1. Get the virus from fridge and mount the pipette on the stereotaxic holder in the right arm.
  2. Position the pipette containing the virus so that its tip touches bregma and calculate the target coordinates.
  3. Remove wet sponge from the craniotomy.
  4. Move the right arm to the target anterior-posterior position. Then start penetration in the brain.
  5. Inject the virus using picospritzer set at 2,000 msec for the period and 1.0 msec for the pulse duration.
  6. Injection is performed in six different points around the target (Figure 2). Two anterior-posterior locations (100 μm anterior to target, 100 μm posterior to target) and three points along the dorsal-ventral axis: 1) 100 μm above the target, 2) target, 3) 100 μm below the target. Inject approximately 1/6 of the viral volume in each point.
    *Note: Wait 5 min between removing the pipette from each anterior-posterior location*.
  7. When injections are finished, pull the arm up. If fiber cannula implantation is to be performed, do not remove the right arm from the stereotaxic frame. Replace the stereotaxic holder with the cannula fiber holder.
  8. Cover craniotomy with a wet sponge in saline.
- Optical fiber implantation
  1. Mount the fiber on the stereotaxic holder in the right arm.
  2. Position the fiber tip on bregma and calculate target coordinates.
  3. Remove wet sponge from the craniotomy and keep it wet with saline (Figure 3A).
  4. In case of additional check for exact fiber location (see Data analysis section), apply a fluorescent dye (*e.g*., DiI) to the fiber sides before implantation, using a syringe with a 30 G needle (be careful and do not cover the tip of the fiber).
  5. Move the right arm to the anterior-posterior target position (Figure 3B).
  6. Insert the fiber slowly into the dorsal-ventral position (Figure 3C). For optogenetic experiments (not for fiber photometry) a 180 μm retraction along the dorsal-ventral axis is recommended for the final target.
    *Note: Before reaching the final target, apply eye ointment in the craniotomy (just enough to cover the space between the fiber and the skull). Alternatively, agarose gel (1%, 10 mg in 1 ml water, Sigma-Aldrich) can be used to cover the craniotomy*.
- Finalization and post-operative care
  1. Apply dental acrylic, in small quantities each time, until fiber implant is firmly fixed to skull.
    *Note: After the acrylic is dry and hard, remove any sharp edges (with the drill if necessary)*.
  2. Suture wound, with approximately two sutures in the back and two in the front.
  3. Remove the fiber cannula holder and place the cap on the optical fiber.
  4. Prepare and apply a mixture of wound powder and 0.3% gentamicin above the sutures and head skin.
  5. Inject 0.5-1 ml of warm sterile saline subcutaneously.
  6. Remove mouse from the stereotaxic frame and let it recover on the heating pad.
  7. Once the mouse is locomoting, transfer it to its home cage.
  8. Monitor the mouse daily for the first four postsurgical days.
  9. After surgery, animals are single housed. Photostimulation or recordings of neural activity with fiber photometry can start 2-3 weeks post-surgery, but if necessary, behavioral training can start 5 days after surgery.

## Data analysis

Viral expression and fiber location can be analyzed using standard histological analysis (please refer to Correia *et al.*, 2017 or Matias *et al.*, 2017 for data examples). To be able to check the exact fiber location, consider applying a fluorescent dye (*e.g*., DiI) to the fiber sides before implantation (be careful and do not cover the tip of the fiber).

## Notes

1. If the virus pipette is clogged before injection, put a saline drop around it and generate some pulses with the picospritzer to unclog it. If this does not work, it might be necessary to perform a fine cut on the tip. In the former option, do not forget to re-mark bregma and adjust the target coordinates.
2. For head-fixed experiments (Matias *et al*., 2017), a head-bar needs to be fixed to the skull. In this case, we recommend applying Super Bond only after the fiber cannula implantation step. Once the fiber cannula is held at the target location, apply Super Bond above the skull and place the head-bar above bregma. Then cover it with more Super Bond and finally, once it is dry, apply dental acrylic above all implants and Super Bond.

## Recipes

1. Super bond C&B
  Use the super bond kit to prepare one small spoon of polymer L-type clear, four drops of monomer and one drop of catalyst. Stir gently and apply immediately (< 2 min) using the brush. It is very important to apply the super bond immediately after preparation. Keep the dispensing dish at 4 °C before using (recommended temperature range of the dish is 10-16 °C) and clean it immediately after usage.
